# Analysis of microRNAs in familial Mediterranean fever

**DOI:** 10.1371/journal.pone.0197829

**Published:** 2018-05-22

**Authors:** Gil Amarilyo, Nir Pillar, Ilan Ben-Zvi, Daphna Weissglas-Volkov, Jonatan Zalcman, Liora Harel, Avi Livneh, Noam Shomron

**Affiliations:** 1 Department of Pediatric Rheumatology, Schneider Children’s Medical Center of Israel, Petach Tikva, Israel; 2 Sackler Faculty of Medicine, Tel Aviv University, Tel Aviv, Israel; 3 Department of Internal Medicine F, Chaim Sheba Medical Center, Tel Hashomer, Israel; University of South Alabama Mitchell Cancer Institute, UNITED STATES

## Abstract

**Objectives:**

Although Familial Mediterranean fever (FMF) is categorized as autosomal recessive, frequent exceptions to this model exist and therefore we aimed to search epigenetic modifications in this disease.

**Methods:**

Ten M694V homozygous FMF patients (the most severe phenotype) were recruited for this study. Patients with inflammatory flare were excluded. Total RNA was extracted from peripheral blood, and microRNA expression profiled using NanoString nCounter technology. These patients were compared to 10 healthy age- and sex-matched controls.

**Results:**

Seven hundred nighty-eight mature human miRNAs were probed, 103 of which had expression levels above the negative control probes. Seven miRNAs showed significant differences in expression in samples from FMF patients compared to healthy controls: four miRNAs were upregulated (miR-144-3p, miR-21−5p, miR−4454, and miR-451a), and three were downregulated (miR-107, let−7d−5p, and miR-148b-3p).

**Conclusion:**

In this pilot study, we identified epigenetic modifications in clinically quiescent FMF patients. More studies are required for exploration of their contribution to FMF pathogenesis and their potential role as clinical biomarkers.

## Introduction

Familial Mediterranean fever (FMF) is the most common Mendelian autoinflammatory syndrome. It occurs most frequently in Sephardic Jewish, Arab, Armenian, Italian, and Turkish populations, with reported carrier frequencies as high as 1:3 to 1:7 [1–3]. FMF is a hereditary disorder caused by gain-of-function mutations in the *MEFV* locus [2]. *MEFV* is comprised of 10 exons encoding the protein pyrin and is expressed primarily in innate immune system cells [1,2]. Pyrin forms a caspase-1-activating inflammasome in response to inactivating modifications of Rho GTPases by various bacterial toxins or effectors [4]. So far, researchers have described more than 130 mutations in *MEFV* [5]. Owing to the autosomal recessive mode of inheritance of the disease, patients would be expected to be homozygous for a single mutation or heterozygous for two different mutations. However, about 30% of patients who are heterozygous for one *MEFV* mutation show a mild inflammatory response that may be manifested by overt disease, including elevated levels of C-reactive protein and serum amyloid A [6]. Indeed, there are reports of families with seemingly dominant inheritance with various penetrance [7,8]. These inconsistencies suggest that epigenetic mechanisms might contribute to the expression of *MEFV* mutations and to the development of overt disease.

MicroRNAs (miRNAs) are short, single-stranded RNA molecules of about 22 nucleotides in length. Their discovery revolutionized our understanding of biologic processes, such as malignant, infectious, and autoimmune mechanisms [9], and contributed to the development of the field of epigenetics. MiRNAs regulate gene expression by base-pair binding to messenger RNA (mRNAs). Depending on the degree of anti-sense complementarity, they can block gene expression via mRNA degradation or inhibition of translation [9].

Recently, Wada *et al*. described changes in the DNA found in the serum of three FMF groups (A. exon 10 mutations, B. exon 3 mutations, and C. absence of exon 3 or 10 mutations) compared to Periodic fever, aphthous stomatitis, pharyngitis and adenitis (PFAPA) controls [10]. However, data regarding miRNA expression in homozygous M694V FMF patients (The most severe FMF phenotype/genotype) remains unknown. The aim of this study, however, was to identify differences in the global miRNA expression profiles in FMF patients homozygous for the M694V mutation (the most severe FMF phenotype/genotype) and in healthy subjects.

## Methods

### Patients and setting

This study was approved by the Chaim Sheba Medical Center Helsinki committee, and was conducted according to the principles expressed in the Declaration of Helsinki. Written informed consents were obtained from the participants. Ten patients diagnosed with FMF according to the Tel-Hashomer criteria [1] were recruited from the rheumatology outpatient clinic of Sheba Medical Center, Tel Hashomer, Israel during routine outpatient follow-up visit between 6/2015-1/2017. All patients were homozygous for the M694V mutation, the most common mutation in FMF, and all had the most severe phenotype of the disease [1]. All patients were in the quiescent phase at the time of the study and were under treatment with colchicine when blood was drawn. Exclusion criteria were a history of chronic disease, acute signs of inflammation at the time of the study, and treatment with glucocorticoids during the past three months. Ten healthy age- and sex-matched volunteers with no positive familial history of FMF served as controls. Written informed consent to participate in the study was obtained from all patients and control subjects.

### MicroRNA extraction and analysis

Total RNA was isolated from peripheral blood mononuclear cells (PBMCs) of patients and controls using TRIzol Reagent (Thermo Fisher Scientific, Waltham, MA, USA) according to the manufacturer’s instructions. Final RNA concentration and purity were measured using a NanoDrop Spectrophotometer (ND-1000; Thermo Scientific).

### MicroRNA expression

The multiplexed NanoString nCounter miRNA expression assay (NanoString Technologies, Seattle, WA, USA) was used to profile 798 human miRNAs. The assay was performed according to the manufacturer’s protocol. Briefly, 100 ng of total RNA was used as input material, with 3 μL of the threefold-diluted sample. Unique DNA tags were ligated onto the 3′ end of each mature miRNA, providing an exclusive identifier for each miRNA species in the sample. Tagging was performed in a multiplexed ligation reaction utilizing reverse complementary oligonucleotide capture probes to ensure hybridization of each miRNA tag to its designated probe. All hybridization reactions were incubated at 64°C for 18 hr. Excess tags were then removed, and the resulting material was hybridized with a panel of fluorescently labeled, barcoded reporter probes specific to the miRNA of interest. miRNA quantities were determined with the nCounter Prep Station by counting individual fluorescent barcodes and measuring the target miRNA molecules present in each sample. Each sample was normalized to the geometric mean of the 100 most highly expressed miRNAs. The mean value of negative controls was set as the lower threshold for each sample. MiRNAs were excluded if at least 50% of their expression value was equal to or below the lower threshold. A total of 103 miRNAs were found eligible for inclusion in subsequent analyses.

### Statistical analysis

All statistical analyses were conducted using R software, version 3.2. Data preprocessing and normalization followed by differential expression analysis were performed using the R package DESeq2 (25516281) and in house scripts. *A priori P* values were adjusted for false discovery rate (FDR) for all of the 103 miRNAs which fulfilled the inclusion criteria.

## Results

We analyzed the miRNA expression profiles of peripheral blood from 10 FMF patients homozygous for the M694V mutation compared to samples from 10 healthy sex- and age-matched controls (Table 1) using a multiplexed direct digital detection and counting system platform. The age, gender, and ethnicity of the two groups are shown in Table 1.

**Table 1:** Demographic data of patients with FMF and matched controls.

Patient/Control no.	Age (years) patients/controls	Gender patients/controls	Patient and control ethnicities*
1	26/24	M/M	Iraq, Morocco
2	26/29	M/M	Morocco, Morocco
3	44/41	M/M	N/A
4	46/42	M/M	Morocco
5	62/67	M/M	N/A
6	20/19	F/F	Tunis, Lybia
7	22/22	F/F	Lybia
8	25/27	F/F	Iraq, Syria
9	25/28	F/F	Iraq, Morocco
10	39/38	F/F	N/A

*All study participants were Jewish.

**Fig 1:**
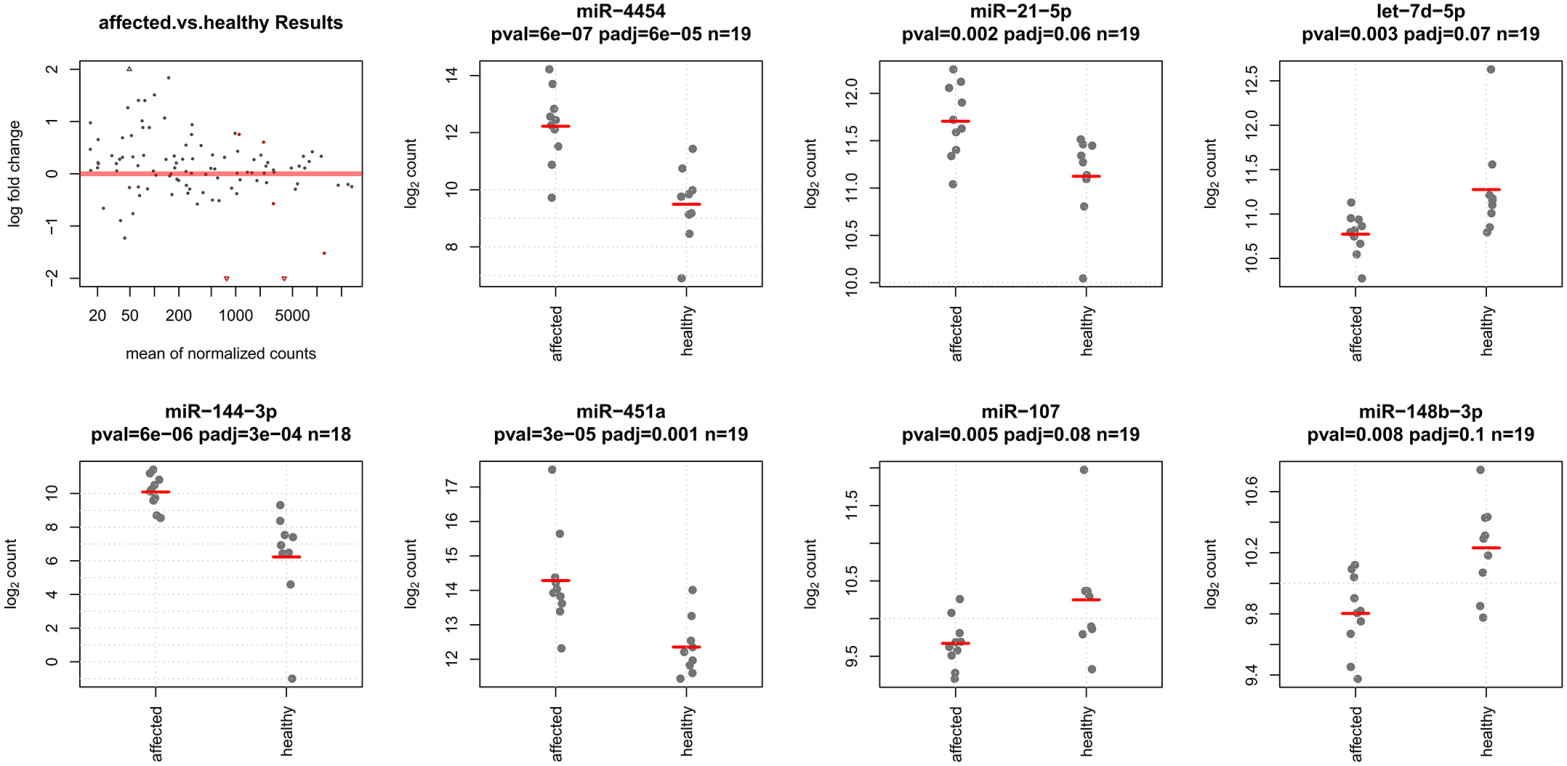
Significant differentially expressed miRNAs. miRNA dispersion analysis demonstrated equal distribution of up- and down-regulated miR transcripts. In FMF patient samples, three miRNAs were downregulated compared to healthy control samples (miR-107, let−7d−5p, and miR-148b-3p), and four miRNAs were upregulated (miR-144-3p, miR-21−5p, miR−4454 and miR-451a).

Of the 798 mature human miRNAs probed, 103 had expression levels above the negative control probes. Seven were found to be significantly deregulated in the patients with FMF: compared to control samples, three were significantly downregulated (miR-107, let−7d−5p, and miR-148b-3p), and four were significantly upregulated (miR-144-3p, miR-21−5p, miR−4454 and miR-451a), all with *P* values of <0.01 (Table 2, Fig 1, and S1 Fig).

**Table 2:** Differentially expressed miRNAs in PBMC from patients with FMF vs. healthy controls and their reported role in immunity.

miRNA (Unique ID)	Expression pattern	Fold change	*P* value	Reported role in immunity	Ref.
hsa-let-7d-5p	Downregulated	0.55	0.003	Preferentially packaged and transferred from Foxp3+ T regulatory (Treg) cells to T helper 1 (Th1) cells, suppressing Th1 cell proliferation and IFN-γ secretion.	[12]
hsa-miR-107	Downregulated	0.47	0.003	Downregulation by toll like receptor 4 in response to activation of murine macrophages with lipopolysaccharide. Downregulation in the inflamed intestines of colitic mice compared to controls, predominantly in epithelial and CD11c (+) myeloid cells, including dendritic cells and macrophages. Decrease in the presence of interleukin (IL)-6, interferon (IFN)-γ, and tumor necrosis factor (TNF)-α, and Increased in the presence of transforming growth factor (TGB)-β.	[13] [14]
hsa-miR-144-3p	Upregulated	13.1	0.000	Positive correlation with IL1-beta levels in lung cancer patients compared to healthy controls.	[15]
hsa-miR-148b-3p	Downregulated	0.65	0.005	Negative regulator of the innate response and antigen presenting capacity of dendritic cells. Inhibition of cytokines production including IL-12, IL-6 and TNF-α. It is therefore thought to act as fine-tuner in regulating the innate response and antigen presenting capacity of dendritic cells, which may contribute to the immune homeostasis and immune regulation.	[16]
hsa-miR-21-5p	Upregulated	1.75	0.003	Upregulation in patients with early psoriatic arthritis or early rheumatoid arthritis.	[17]
hsa-miR-4454	Upregulated	12.8	0.000	upregulation by the transcription factor NF-κB Positive correlation with disease activity in ulcerative colitis.	[18] [19]
hsa-miR-451a	Upregulated	4.36	0.000	Upregulation in autoimmune diseases such as rheumatoid arthritis and Systemic Lupus Erythematosus. Downregulation correlates with increased secretion of IL-6, TNF, CCL5/RANTES, and CCL3/MIP1α in influenza infected dendritic cells. miR-451 levels were themselves increased by IL-6 and type I interferon, potentially forming a regulatory loop.	[20] [21]

**Fig 2:**
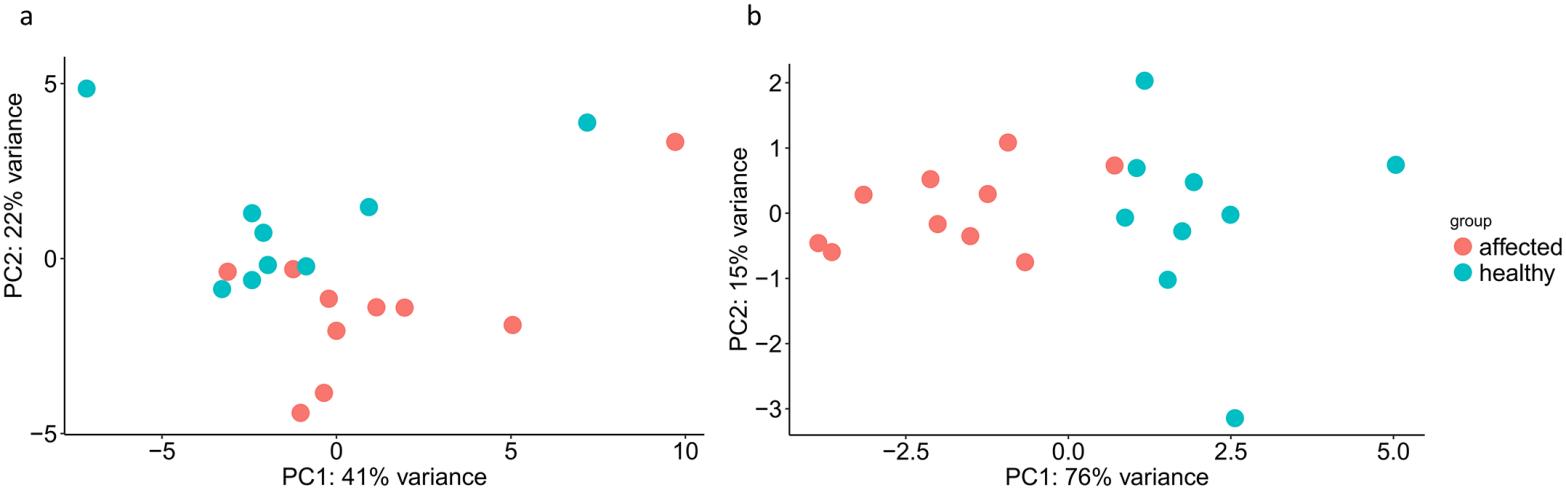
PCA analysis of miRNAs. (A) PCA analysis of 103 miRNAs with reasonable expression levels demonstrated a clear separation between the FMF and control groups. (B) PCA analysis of 7 differentially expressed miRNAs revealed distinguishable profiles of the FMF and control groups.

To ensure that the observed changes were true biological effects and not technical artifacts, expression of two of the differentially expressed miRNAs was validated in FMF and healthy control samples using Taqman quantitative real time polymerase chain reaction (qRT-PCR). Each miRNA was quantified in each sample, and its expression level was normalized to the reference RNA, U6-snRNA. In both groups, miRNA expression patterns were consistent with the NanoString results (R^2^ = 0.93) (S2 Fig). PCA analysis of 103 miRNAs with reasonable expression level presented a clear separation between the FMF and the control group (Fig 2A). PCA analysis of the 7 differentially expressed miRNAs demonstrated complete separation between the FMF and control group (Fig 2B).

Next, we sought to determine if the differentially expressed miRNAs are known to be involved in an immune response. We considered all miRNAs appearing in InnateDB, a comprehensive manually-curated knowledgebase of mammalian innate immunity, as innate miR [11]. Of the total 49 immune miRNAs that appear in the database, three (miR-107, miR-21 and miR-148) were differentially expressed in our cohort. This number was significantly higher than expected by chance (*P* = 0.003).

## Discussion

This is the first study reporting thorough miRNA screening in patients with the most severe FMF phenotype/genotype. By utilizing miRNA-based multiplex analysis followed by qRT-PCR validation, we identified seven miRNAs with statistically significant (*P-value*<0.01) differential expression between patients with FMF and healthy controls. Patients with acute inflammation were excluded from all analyses in order to avoid masking miRNAs involved in this process. All patients were under treatment with colchicine when blood was drawn.

We found that miR-107, let−7d−5p, and miR-148b-3p were downregulated in patients with FMF, and miR-144-3p, miR-21−5p, miR−4454, and miR-451a were upregulated. Importantly, all of these miRNAs were found to be involved in immune processes (Table 2). Moreover, miR-107, miR-21 and miR-148 were found in InnateDB, which integrates interaction and pathway information from several of the major publicly available databases and aims to capture an improved coverage of the innate immunity interactome. Interestingly, miR-107 and miR-148 were reported to serve as negative regulators of the innate immune system, whereas miR-21 plays a proinflammatory role. In this study, we showed that miR-107 and miR-148 are downregulated in FMF patients, whereas miR-21 is upregulated, demonstrating an overall proinflammatory profile of the innate immune system of FMF patients, even in the quiescent phase.

Although major progress has been made in revealing the genetic basis and pathogenesis of FMF, the mode of inheritance is still not fully understood. Therefore, FMF continues to be diagnosed primarily by clinical criteria, with genetic testing serving solely for confirmation [1]. Our study expands the already known genetic basis of FMF and sheds light on the complex mode of inheritance which cannot be explained by genetic mutations alone. Utilizing PCA analysis, we showed that the seven differentially expressed miRNAs we detected can be used to differentiate FMF patients from the matched control group. This separation was not detected in PCA analysis of all 103 significantly expressed miRNAs, suggesting that our findings may serve as the basis for the development of a novel biomarker, which will increase the sensitivity and specificity of these genetic tests and expand prognostic information. Only one miRNA, miR-451a, was found to corroborate the results of Wada *et al*. This difference may be explained by the difference in patient selection and biological samples tested.

This study was limited by the small sample size, although between-group differences achieved statistical significance. In addition, we restricted the study sample to patients with the most severe form of FMF (M694V homozygous mutation) in order to maximize the potential differences from controls. Therefore, our findings may not be relevant to other forms or genetic mutations of the disease. Finally, all of our patients were successfully treated with colchicine, which may have an impact on miRNA expression.

Further research is warranted in order to elucidate critical FMF manifestation such as susceptibility to early disease, disease severity, risk of the development of amyloidosis, and resistance to colchicine. All these factors might be ultimately explained, at least in part, by epigenetic modifications.

## Supporting information

S1 Fig miRNA expression heatmap of FMF patients vs. healthy controls. Expression profiles of microRNAs in PBMCs from homozygous M694V quiescent FMF patients (light blue) and age- and sex-matched healthy controls (black).
(JPG)

S2 Fig Validation of Nanostring analysis by qPCR. Two differentially expressed microRNAs—let-7d and miR-144 were analyzed by qPCR, and the results compared to NanoString results, revealing a correlation of 0.93 between the modalities.
(PNG)
