# Supplementary figures and images for: Analysis of microRNAs in familial Mediterranean fever
Source: PLoS One. 2018 May 22;13(5):e0197829. doi: 10.1371/journal.pone.0197829 (PMC5963758; doi:10.1371/journal.pone.0197829)

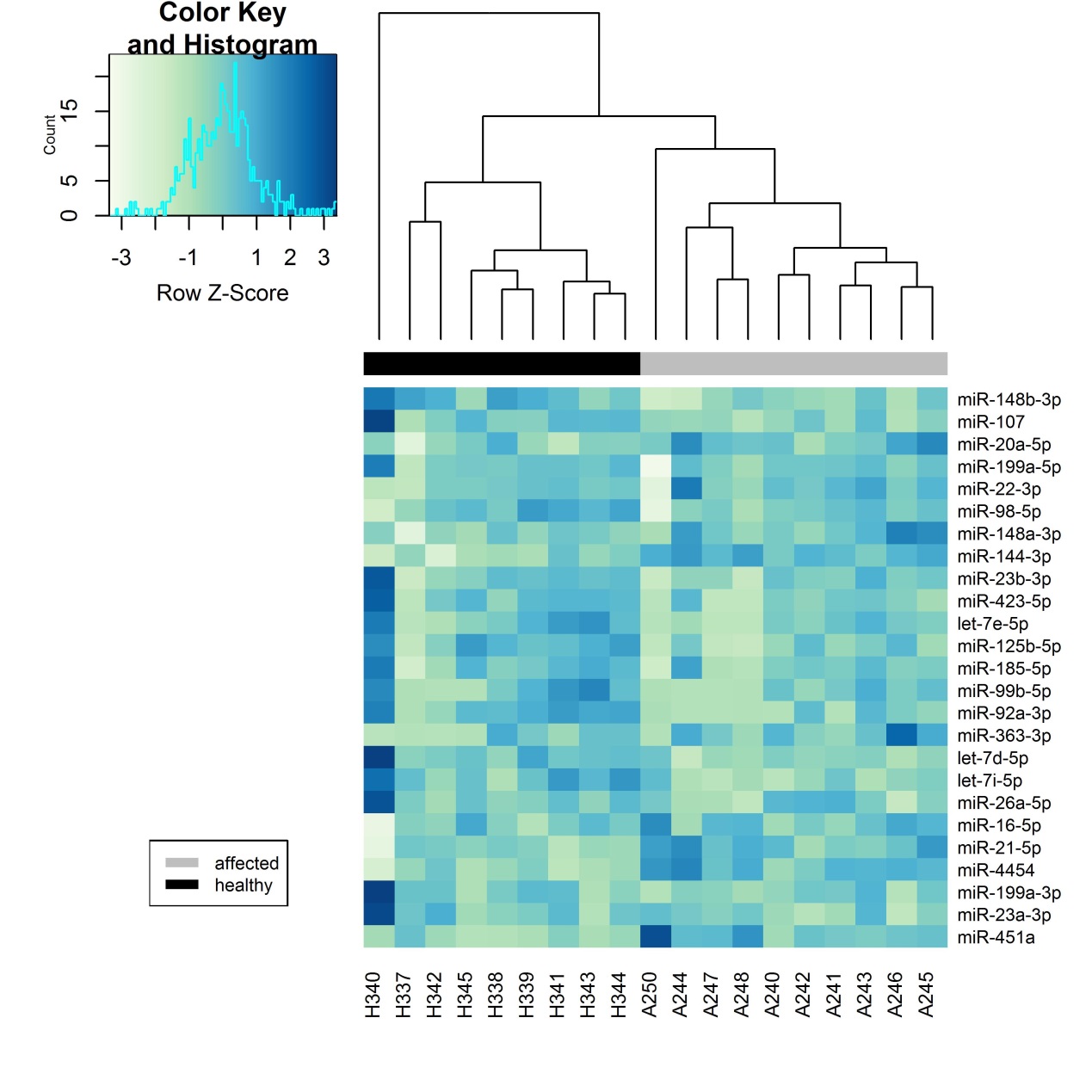

Supplement: S1 Fig — Expression profiles of microRNAs in PBMCs from homozygous M694V quiescent FMF patients (light blue) and age- and sex-matched healthy controls (black). (JPG) [file pone.0197829.s001.jpg]

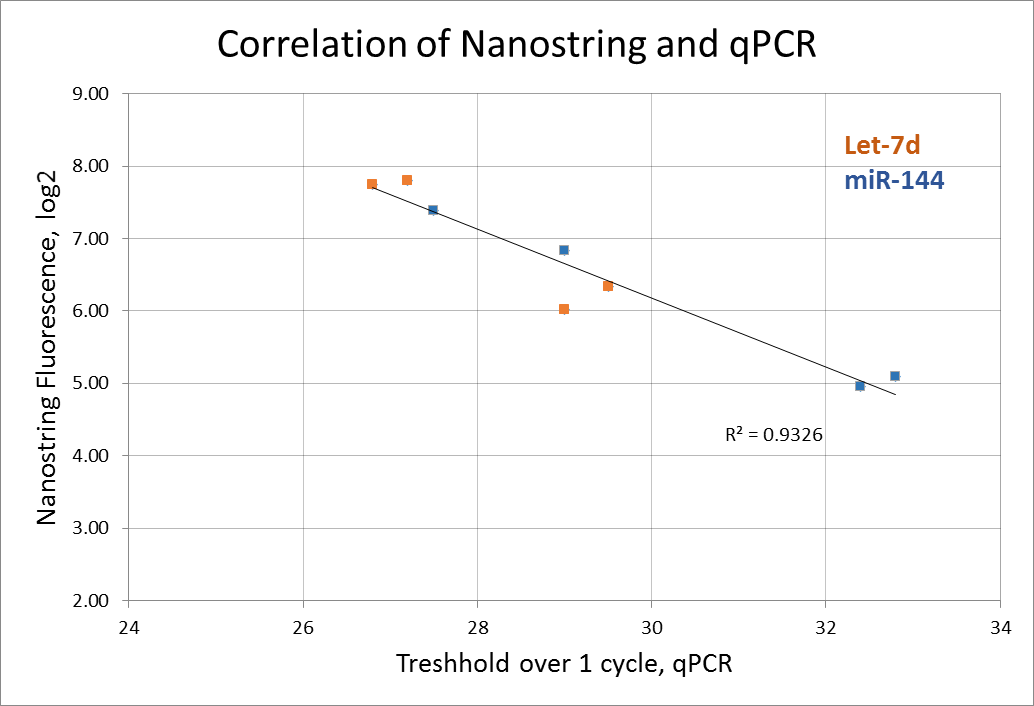

Supplement: S2 Fig — Two differentially expressed microRNAs—let-7d and miR-144 were analyzed by qPCR, and the results compared to NanoString results, revealing a correlation of 0.93 between the modalities. (PNG) [file pone.0197829.s002.png]
